# Economic evaluations of interventions focusing on child abuse and neglect in high-income countries: a systematic review

**DOI:** 10.3389/fpsyt.2023.1031037

**Published:** 2023-06-21

**Authors:** Tom Kugener, Isabell Wiethoff, Ghislaine van Mastrigt, Bram van den Berg, Silvia M. A. A. Evers

**Affiliations:** ^1^Department of Health Services Research, Care and Public Health Research Institute (CAPHRI), Maastricht University, Maastricht, Netherlands; ^2^Department of Quality, Policy and Monitoring, Nederlands Jeugdinstituut, Utrecht, Netherlands; ^3^Centre for Economic Evaluation and Machine Learning, Trimbos Institute, Netherlands Institute of Mental Health and Addiction, Utrecht, Netherlands

**Keywords:** child abuse and neglect, maltreatment, economic evaluation, cost-effectiveness, review-systematic

## Abstract

**Introduction:**

Child abuse and neglect are together considered to be an important public health problem with a high individual and societal burden. Different interventions have been developed to prevent, diagnose, or treat maltreatment. While their effectiveness has been synthesized in prior reviews, the analysis of their cost-effectiveness is less common. The aim of this study is to synthesize and analyse economic evaluations of interventions focusing on child abuse and neglect in high-income countries.

**Methods:**

A systematic literature review was performed using MEDLINE, EMBASE, EconLit, PsycInfo and NHS EED. This study follows the PRISMA guidelines and double scoring was performed. The review includes trial- and model-based economic evaluations of preventive, diagnostic, and treatment related interventions in children up to 18 years or their caregivers. Risk of bias was assessed using the CHEC-extended checklist. The results are presented in a cost-effectiveness plane.

**Results:**

Of 5,865 search results, the full texts of 81 were analyzed, resulting in the inclusion of 11 economic evaluations. Eight of the included studies focus on prevention of child abuse and neglect, one study on diagnosis, and two on treatment. The heterogeneity between studies did not allow for the quantitative pooling of results. Most interventions were cost-effective, with the exception of one preventive and one diagnostic intervention.

**Conclusion:**

This study was subject to some limitations, as no gray literature was included, and the selection of studies may have been arbitrary due to varying terminologies and methodologies in the field. However, the quality of studies was high, and several interventions showed promising results.

**Systematic review registration:**

https://www.crd.york.ac.uk/prospero/display_record.php?ID=CRD42021248485, identifier: CRD42021248485.

## Introduction

Child abuse and neglect is highly prevalent in high-income countries, having a great impact on the child and their surroundings, consequently leading to a high burden on society. According to a review by Gilbert et al. (1) 4–16% of children in high-income countries experience physical abuse yearly, and 10% experience neglect or psychological abuse. The cumulative prevalence for sexual abuse of children ranges between 5% and 30% (1). Current estimates by the Centers for Disease Control and Prevention (CDC) show similar results. The CDC (2) estimates that in 2020, one in seven children in the US experienced child abuse or neglect. Maltreatment prevalence rates are expected to be similar for various high-income countries, such as the US, Canada, and European countries (3). A vast number of studies have established an association between maltreatment and different adverse outcomes, including an increased risk for several physical and mental health conditions, emotional and functional impairment, lower wellbeing, and higher risk of delinquent behavior (3–5).

Besides the individual burden, child maltreatment represents a global public health issue with high economic and societal costs (3, 4). Based on several studies, the United Nations estimates that the global burden of violence against children ranges between 2 and 10% of the global Gross Domestic Product (GDP) (6). According to the European Commission, in European countries the annual economic burden of maltreatment represents 4% of the GDP (3). These estimates include costs related to child welfare services, educational services, criminal justice services and productivity losses, in addition to healthcare costs (3).

The current article focuses on economic evaluations of interventions in child maltreatment or child abuse and neglect. The latter terms, i.e., child maltreatment or child abuse and neglect, will be used interchangeably throughout the article. Child maltreatment or child abuse and neglect are defined in this study as “all forms of physical and/or emotional ill-treatment, sexual abuse, neglect or negligent treatment or commercial or other exploitation, resulting in actual or potential harm to the child's health, survival, development or dignity in the context of a relationship of responsibility, trust or power” (7). Four types of child abuse or neglect are commonly distinguished in the literature, namely physical abuse, emotional abuse, sexual abuse, and neglect (7, 8). The definitions of the different types of abuse and neglect can be found in the World Health Organization's (WHO) 1999 Report of the consultation on child abuse prevention (7).

The high burden of child abuse and neglect has led to the development of different interventions to prevent the maltreatment of children or adolescents and, in case of prior maltreatment, provide them with adequate help and treatment. Even though the effectiveness of child abuse and neglect related interventions has been extensively covered in prior reviews, the economic evaluation of these interventions has been given less consideration (9–11). Economic evaluations can be defined as “the comparative analysis of alternative courses of action in terms of both their costs and their consequences” (12). There are four types of full economic evaluations, namely cost-minimization analysis (CMA), cost-effectiveness analysis (CEA), cost-utility analysis (CUA) and cost-benefit analysis (CBA) (12, 13). In each of these types of economic evaluations, the costs and effects of two or more interventions are analyzed and compared. While the costs are analyzed in the relevant monetary unit or currency, the types of outcomes vary across the types of economic evaluations, ranging from monetary outcomes to clinical effectiveness or quality of life (12, 14). In a CMA, the outcomes for both interventions are the same and their costs are compared. In a CEA, clinical outcomes or assessments from validated tools, as well as their costs are compared. A CUA includes quality adjusted life years as outcome and in a CBA benefits are measured in monetary units (12).

The current literature reveals that several interventions to either prevent, treat, or diagnose child maltreatment have been developed. Some existing reviews have analyzed the cost-effectiveness of such interventions (9, 15, 16). According to Dalziel and Segal (9) the cost-effectiveness of home-based interventions varies strongly, and is highest for more complex interventions targeting high-risk populations through professionals from different disciplines. El-Banna et al. (15) conclude that most social care interventions seem cost-effective but highlight the lack of standardized procedures or methods for analyzing the cost-effectiveness of such interventions. Peterson and Kearns (16) came to a similar conclusion, analyzing violence prevention interventions.

Existing reviews, however, mainly differ from the current review in three aspects. First, they may be outdated, as they include relatively old studies conducted prior to 2010 (9). Second, they focus on a broader range of interventions, related to, e.g., general violence prevention and social care interventions (15, 16). Finally, the systematic assessment of the quality of individual economic evaluations is given less attention or is not reported.

The preliminary review of the literature consequently shows the necessity of an up-to-date review, focusing on economic evaluations of child maltreatment interventions in high-income countries conducted after 2010. Accordingly, the aim of this study is to analyze the current evidence on the cost-effectiveness of various interventions focusing on the prevention of child abuse and neglect or services aimed at children and adolescents who have experienced abuse or neglect.

## Methods

To analyze the current evidence on economic evaluations of relevant interventions in high- income countries, a multipurpose systematic literature review of model- and trial-based economic evaluation was performed. A systematic review was considered appropriate to ensure a systematic and reproducible collection, analysis, and synthesis of relevant primary studies. A multipurpose systematic review was chosen, as the primary goal of the study is to identify knowledge gaps and inform policy decisions (17). The pre-specified methods follow the Preferred Reporting Items for Systematic Reviews and Meta-Analyses (PRISMA) guidelines (18) and the 5-step approach proposed by Van Mastrigt et al. (17). Furthermore, a protocol of this review (CRD42021248485) has been published in the International Prospective Register of Systematic Reviews (PROSPERO) and can be found in the Supplementary material (https://www.crd.york.ac.uk/prospero/display_record.php?ID=CRD42021248485). The study selection as well as the data extraction and quality appraisal were assessed by two researchers independently. Initial disagreements were discussed between the researchers and a third researcher was consulted to reach consensus if needed.

### Data collection/literature search

A literature search was performed using MEDLINE, EMBASE, EconLit, PsycInfo, and NHS EED. As NHS EED, a database focusing on economic evaluations in health care, is no longer publishing, only publications up to March 2015 could be included from this source. Furthermore, the references from included studies were analyzed and a citation search was performed. The search strategy was constructed based on the following keywords related to the research aim: “Youth,” “Economic Evaluations,” “cost-of-illness” and “child abuse and neglect (interventions).” The search strategy includes “cost-of-illness” studies, as this article is part of a larger project by Maastricht University, in collaboration with the Dutch Youth Institute, looking at the economic impact of child abuse and neglect. Furthermore, the Pediatric Economic Database Evaluation (PEDE) was checked for additional, relevant studies.

As the development of a valid new search strategy is time-consuming, existing verified search filters were retrieved through the InterTASC Information Specialists' Sub-Group Search Filter Resource. In general, search filters with high sensitivity are most desirable for a systematic review of economic evaluations (19). The chosen Youth-related keywords were based on two search filters from the Canadian Health Libraries Association for children and adolescents. For “economic evaluations,” a search filter from the Canadian Agency for Drugs and Technologies in Health (CADTH) was included. For “Child Abuse & Neglect (interventions),” the search strategy from El-Banna et al. (15) was adapted to the aim and search strategy of this review. Finally, a conceptual approach was applied to establish a search filter to retrieve cost-of-illness studies.

Synonyms for one concept were combined through the Boolean operator “OR,” while different concepts were combined through the Boolean operator “AND.” The search strategy was adapted individually for each database, as the transferability of database-specific search filters is often limited (19). The literature search for all databases was performed on 4 May 2021. To manage and analyze the search results, EndNote (version X9.3.3) was used as reference software. Further details on the final search strategy for the different databases can be found in the Supplementary material. Reference checking and citation tracking was performed to identify studies which may have been missed through the search strategy.

### Inclusion/exclusion criteria

The eligibility of retrieved studies was assessed based on the following inclusion and exclusion criteria. To be included, studies had to include a full economic evaluation (EE). Full economic evaluations are defined as a comparison between two or more programs or interventions in terms of both costs and effects or benefits (17). To include all the relevant literature, model-based and trial-based EEs were included. Second, to be included, studies had to focus on interventions for children and adolescents aged 0 to 18, according to the definition of child abuse and neglect (CAN) by the WHO, and/or interventions for their caregivers. Third, relevant studies had to include an intervention specifically focusing the prevention of CAN or services provided to children or their family after abuse or neglect occurred. To be included, interventions had to focus on either the prevention, diagnosis or treatment of children at risk of or experiencing abuse and neglect. Studies focusing on interventions not specifically addressing CAN or focusing on mental health conditions in parents or children were excluded. Only studies performed in a high-income country (based on the World Bank Atlas Method Classification in 2020) were included, as the rates of CAN and the interventions used in these countries are expected to be comparable. To ensure the inclusion of the most recent evidence, studies published prior to 2010 were excluded, as well as studies written in languages other than English, German, and French. Conference abstracts, editorials and letters were excluded. Although systematic reviews were excluded, their references were analyzed for further results.

### Data extraction and quality assessment

An extraction sheet for systematic reviews of economic evaluations was constructed based on the 35 items described in Wijnen et al. (20). To ensure a systematic application of the extraction sheet, a picklist was constructed in Excel (version 2101). The results of the data extraction were summarized in two tables: one focusing on the study characteristics and the second focusing on the study results.

As the review includes model- and trial-based EEs, the extended Consensus Health Economic Criteria (CHEC) checklist has been applied to critically assess eligible EEs, as it is recommended by Cochrane (21). Furthermore, it is the only consensus based quality assessment tool (20). The CHEC-extended checklist includes 20 items or questions which can be answered by Yes, No or Suboptimal and scored to assess the methodological quality of full EEs. To adequately implement the checklist and increase transparency, the assessment instructions provided by Maastricht University were applied (https://www.maastrichtuniversity.nl/research/caphri/our-research/creating-value-based-health-care/chec-list-consensus-health-economic). The results of the risk of bias assessment were summarized in a table enabling the ranking of the studies based on their quality. The CHEC-extended checklist can be found in the Supplementary material.

All monetary units were adapted to a single currency and reference year using a tool provided by the Campbell and Cochrane Economics Methods Group (CCEMG) and the Evidence for Policy and Practice Information and Coordinating Center (EPPI-Center) (https://eppi.ioe.ac.uk/costconversion/default.aspx). Consequently, the values for benefit-cost estimates expressed as a ratio between benefits and costs do not change. The analysis of included studies was clustered according to the type of intervention. The results of all included studies were visualized in a cost-effectiveness plane with a fixed threshold for the willingness to pay per gain of quality-adjusted life-year (QALY). A threshold of 20,000 Euros per QALY was chosen based on the most conservative cost-effectiveness threshold value in the Netherlands (22).

## Results

**Figure 1:**
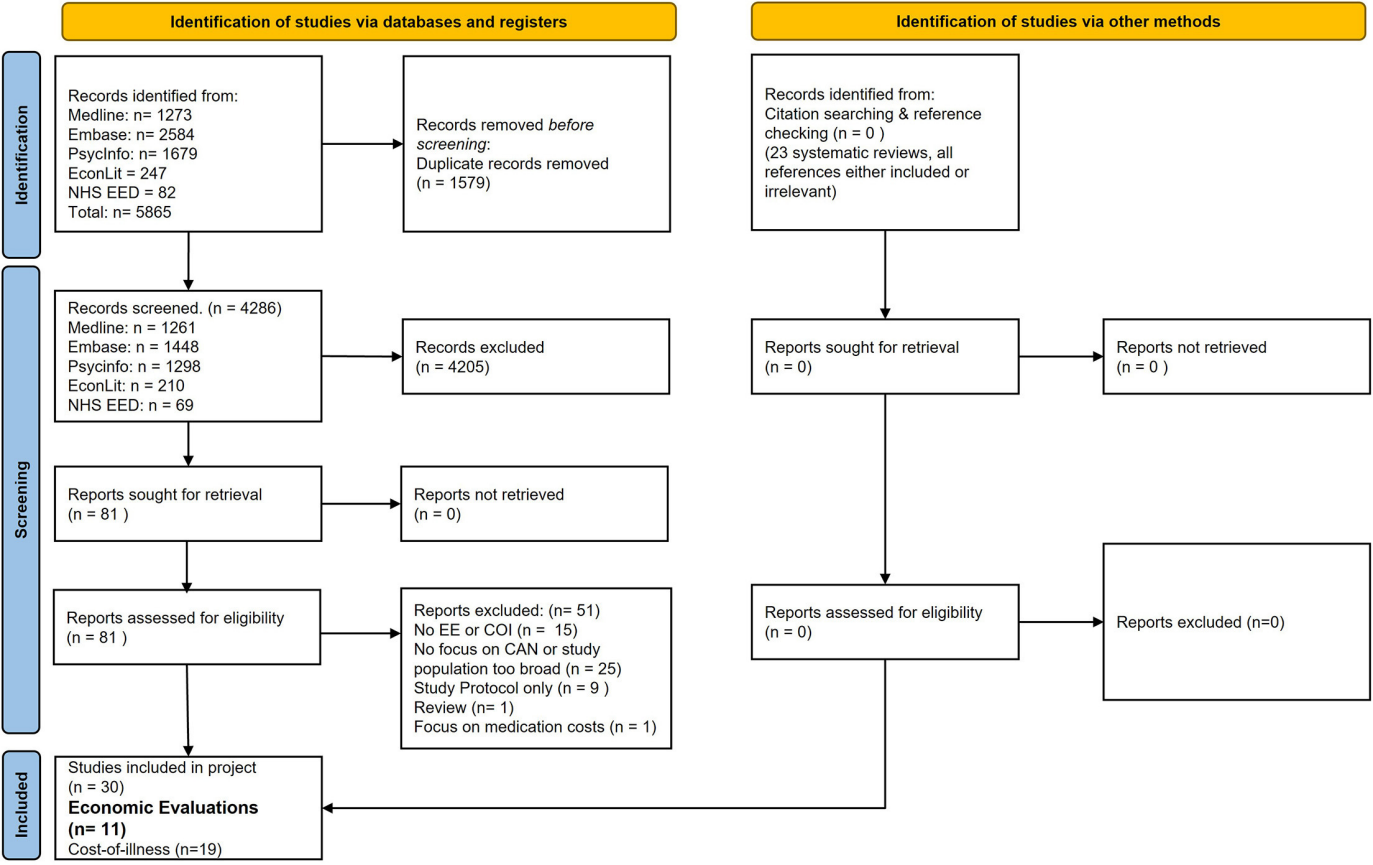
PRISMA flowchart (18).

As can be seen in Figure 1 a total of 5,865 studies were retrieved through the previously established search strategy. After deduplication (*n* = 1,579), 4,286 studies remained eligible for initial screening. After scanning the title and abstract of these studies, 4,205 studies were excluded, and 81 studies remained eligible for full-text screening. The main reasons for exclusion in abstract scanning were the focus on lower income countries or on the prevention or treatment of specific mental health conditions in children and their caregivers. The full-text analysis of the remaining studies resulted in the inclusion of 11 economic evaluations and 19 cost-of-illness studies. In this article, only the retrieved economic evaluations are analyzed, while the cost-of-illness studies will be covered in a second article. The main reasons for exclusion in the full-text analysis were a study design which included neither a cost-of-illness nor an economic evaluation (n = 15), a focus on other things than maltreatment, including conditions or events such as depression, anxiety, self-harm, conduct disorder or behavioral disorders (*n* = 25). While similar interventions were identified in the studies focusing on the mentioned conditions or events, the studies focused on other outcomes not related to abuse and neglect. Nine articles were excluded as they included only the study protocol for economic evaluations. Finally, one study focused on medication costs only and one study included a review. The abstracts of identified systematic reviews were analyzed. Twenty-three systematic reviews were considered potentially relevant based on their abstract, and their references were screened for relevant economic evaluations. The analysis of references to relevant systematic reviews did not lead to the inclusion of any further studies. All checked references were either included already or did not fit the inclusion criteria. Other reviews did not include any relevant studies and were not further analyzed after scanning their abstracts. No additional relevant studies were identified through the PEDE database, as they were either already included, or did not meet all the inclusion criteria (conducted prior to 2010).

### General characteristics

Of the 11 studies deemed eligible, 4 studies include a trial-based EE (23–26), while 7 studies focus on model-based EEs (27–33). Regarding the type of economic analyses, 4 studies produced a cost-utility analysis (23, 24, 30, 31), 5 studies a cost-benefit analysis (26, 27, 29, 32, 33), and 6 studies a cost-effectiveness analysis (23–26, 28, 33). Two studies included both a cost-effectiveness and a cost-utility analyses (23, 24) and two studies included a cost-effectiveness and cost-benefit analysis (26, 33). However, QALYs in children were assessed in only two studies (30, 31). This may be due to the complications associated with determining QALY scores in young children. Five of the cost-effectiveness studies include prevented maltreatment cases as outcome (23–26, 33), while one study focuses on additional convictions (28). The CEA focusing on avoided cases of maltreatment and the CBA were the most applied types of economic evaluations. CBA may be beneficial, as the broad range of consequences of maltreatment and possible effects of interventions can be summarized in a single monetary value.

Five studies were performed in the United States (26, 28, 29, 32, 33), two in Australia (25, 31), two in the United Kingdom (23, 24), one in New Zealand (30) and one in Canada (27). Two studies applied a UK NHS and personal social services perspective (23, 24) and four studies a societal perspective (23, 25, 27, 33), with some studies additionally including more narrow perspectives. Finally, two studies applied a participant, taxpayer and society perspective based on the Washington State Institute for Public Policy (WSIPP) model for cost-benefit analyses (29, 32). For three studies the perspective had to be assumed, as it was not specifically mentioned (26, 28, 30). Eight studies focused on the prevention of maltreatment before or after first contact with child protection services (23–27, 30, 32, 33), one on diagnosis (28), and two studies on the treatment of CAN (29, 31). The age of included children ranged from birth to 17 years of age.

The analysis of the results of individual studies is divided according to the type of intervention. Preventive interventions, which account for most of the included studies, are analyzed first, followed by EEs of diagnostic- and treatment-related interventions. The general characteristics of all included studies can be seen in Table 1.

**Table 1:** Characteristics of included studies.

**Authors (year)**	**Participants**	**Perspective**	**Type of economic Evaluation and Intervention**	**Country**	**Trial- or model-based**	**Comparator**	**Outcome measure**	**Conclusion**
Barlow et al. (23)	Children under 2.5 years and parents in substance abuse treatment	a) NHS and Personal social services b) Societal	CEA: *Parents under Pressure* (Prevention)^*^	UK	Trial	Treatment as usual	QALY & child abuse potential	Cost-effective
Barnes et al. (24)	Expectant mothers	NHS and Personal social services	CEA: *Group Family Nurse Partnership* (Prevention)^*^	UK	Trial	Usual Care	QALY, child abuse potential & maternal sensitivity	Not cost-effective
Beaulieu et al. (27)	Based on children aged 0-24 months	a) Societal b) Health services perspective	CBA: *PURPLE* program (Prevention)	Canada	Model	No program/Period before intervention	Monetary outcomes	Dominant
Block et al. (28)	Possibly abused children	Societal assumed	CEA: *Multiple Interviews* (Diagnosis)	US	Model	Single interviews	Additional convictions	Unclear
Dalziel et al. (25)	Children aged 2-8 years and parents in methadone treatment	Societal	CEA: *Parents Under Pressure* (Prevention)	Australia	Trial	Usual care and brief intervention	Prevented cases of maltreatment	Cost-effective
Dopp et al. (29)	Children aged 10–17 (with determination by CPS that CAN occurred)	Participant, taxpayer and society (WSIPP model)	CBA: *Multisystemic Therapy* for child abuse & Neglect (Treatment/ Prevention after reported abuse)	US	Model	Enhanced outpatient treatment	Monetary values	Cost-effective
Friedman et al. (30)	National Births	Societal assumed	CUA: Shaken Baby Prevention program (Prevention)	New Zealand	Model	No treatment comparator	QALY	Dominant
Gospodarevskaya and Segal (31)	Based on 10-year-old baseline cohort with PTSD due to sexual abuse	Mental healthcare system a) 12-month timeframe b) 31 years' timeframe	CUA: *TF-CBT, TF-CBT and SSRI, and Non-Directive Supportive Counseling* (Treatment)	Australia	Model	No treatment comparator	QALY	Non-directive counseling: (Least) cost-effective TF-CBT (& SSRI): Cost-effective
Kuklinski et al. (32)	Children aged 10 to 24 months	Participant, Taxpayer and Society (WSIPP model)	CBA: *Promoting First Relationships* (Prevention after open CPS report)	US	Model	Resource and/or referral	Monetary outcome	Cost-effective
Lane et al. (26)	Children below 6 years old	Health care assumed	CEA: *Safe Environment for every Kid (SEEK)* (Prevention)^**^	US	Trial	Routine pediatric care	Prevented cases of maltreatment	Cost-effective
Peterson et al. (33)	Based on hypothetical cohort estimated for each US state	a) Government payer b) Societal	CEA: *Child Parent Centers (CPC)* and *Nurse*−*FamilyPartnership*(*NFP*)^**^	US	Model	Control groups from prior studies	Prevented cases of maltreatment	CPC: Dominant (less than NFP) NFP: Dominant

^*^Studies additionally include outcomes of a cost-utility analysis.

^**^Studies additionally include outcomes of a cost-benefit analysis.

### Quality of the identified studies

Applying the CHEC-extended checklist for the quality of economic evaluations, the average quality of included studies was 89.21%. The lowest score was 58.3% (28). The highest score of 100% was achieved by three studies (24, 29, 31). The other studies all had scores ranging between 82.5% and 97.4%. No direct trends or associations between study characteristics or outcomes and quality scores were observed.

As can be seen in Table 2, most points were deducted for “Q7”, “Q8” and “Q15”. A complete list of the questions can be found in the Supplementary material. “Q7” is related to the chosen perspective. Points were deducted as some studies did not specifically mention the applied perspective, which consequently had to be assumed (26, 28, 30). “Q8” focuses on the inclusion and reporting of relevant costs for both the intervention of interest and the comparator. The deduction of points was caused by a lack of transparency in reporting costs for both alternatives or for missing costs that should be included, considering the chosen perspective (23, 25–28, 30). “Q15” asks whether costs and outcomes are discounted properly. In some studies, the discount rate was not reported or not applied to all relevant costs and outcomes (26, 28, 33).

**Table 2:** Quality assessment based on the CHEC-extended checklist.

**Author(s) and year**	**Q1 Study Population described**	**Q2 Alternatives described**	**Q3 Well-defined research question**	**Q4 Appropriate study design**	**Q5 Structural assumptions & validation**	**Q6 Appropriate time horizon**	**Q7 Appropriate perspective**	**Q8 Relevant costs for both alternatives**	**Q9 Costs measured appropriately**	**Q10 Costs valued appropriately**	**Q11 Relevant outcomes for both alternatives**	**Q12 Outcomes measured appropriately**	**Q13 Outcomes valued appropriately**	**Q14 Incremental analysis of costs and outcomes**	**Q15 Future costs and outcomes discounted**	**Q16 Sensitivity analysis performed**	**Q17 Conclusion supported by data**	**Q18 Generalizability discussed**	**Q19 Potential conflict of interest**	**Q20 Ethical issues discussed**	Total*(%)
Barlow et al. (23)	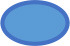	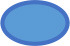	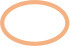	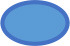	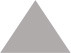	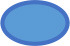	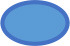	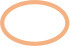	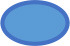	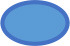	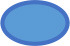	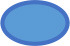	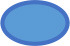	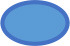	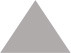	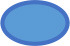	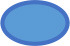	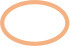	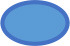	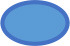	91.7
Barnes et al. (24)	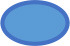	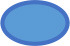	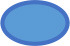	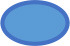	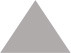	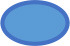	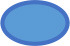	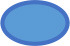	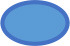	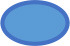	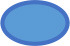	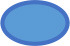	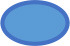	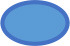	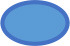	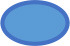	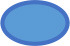	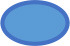	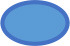	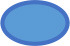	100
Beaulieu et al. (27)	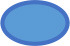	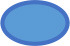	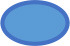	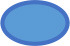	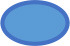	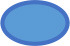	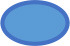	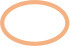	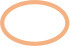	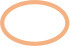	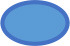	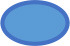	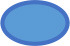	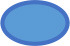	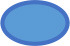	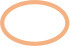	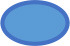	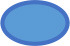	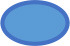	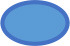	90.0
Block et al. (28)	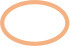	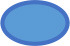	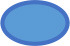	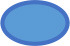	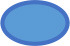	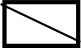	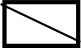	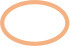	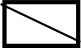	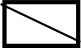	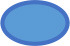	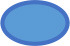	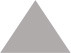	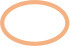	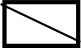	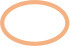	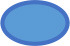	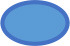	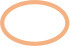	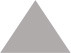	58.3
Dalziel et al. (25)	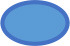	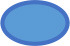	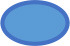	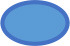	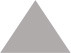	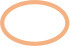	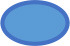	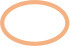	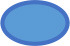	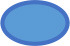	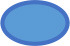	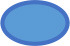	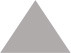	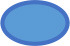	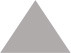	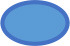	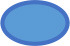	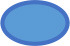	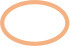	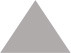	90.6
Dopp et al. (29)	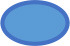	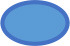	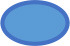	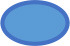	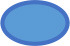	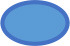	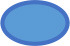	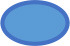	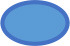	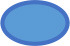	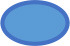	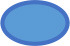	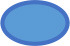	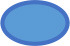	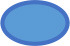	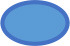	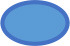	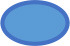	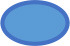	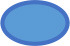	100
Friedman et al. (30)	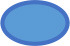	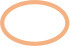	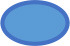	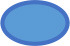	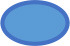	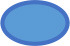	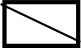	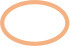	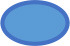	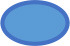	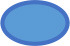	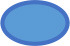	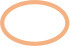	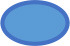	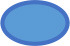	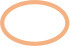	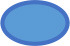	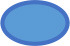	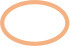	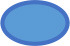	82.5
Gospodarevskaya and Segal (31)	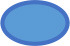	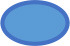	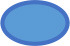	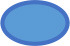	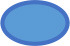	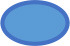	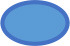	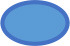	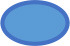	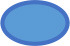	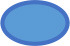	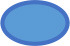	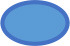	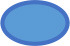	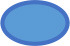	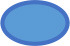	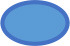	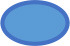	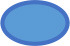	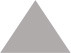	100
Kuklinski et al. (32)	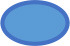	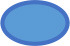	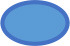	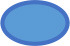	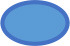	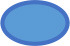	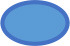	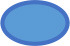	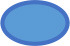	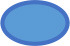	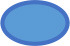	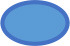	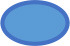	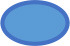	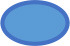	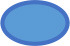	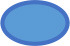	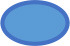	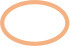	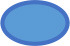	**97.4**
Lane et al. (26)	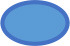	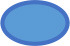	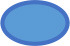	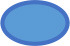	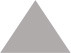	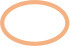	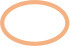	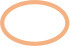	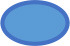	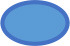	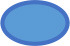	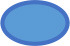	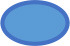	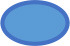	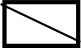	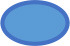	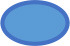	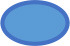	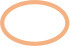	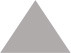	**83.3**
Peterson et al. (33)	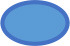	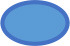	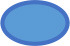	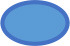	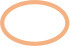	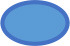	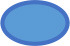	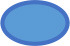	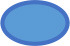	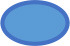	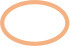	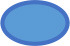	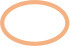	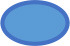	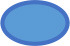	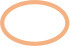	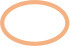	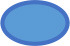	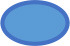	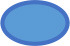	**87.5**
Total^*^ (%)	95.5	95.5	95.5	100	92.9	81.8	77.3	72.7	86.4	81.8	100	95.5	94.4	95.5	72.2	81.8	100	90.9	81.8	100	

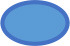
 YES – 1 point given, 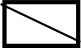 NO – 0 points given, 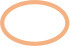
Suboptimal (SO) – 0.5 points given, 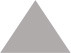
Not applicable.

^*^ Total (%) = $\frac{Number\;of\;YES^{*}1\;+\;Number\;of\;SO^{*}0.5}{\left(Number\;of\;YES\;+\;Number\;of\;SO\;+\;Number\;of\;NO\right)}$ ^*^100.

## Results of the included study

### Preventive interventions

#### Parents under pressure

Barlow et al. (23) analyze the cost-effectiveness of the *Parents Under Pressure* program compared to usual care from a UK NHS and personal social services and from a societal perspective. *Parents under Pressure* is a mainly home-based intervention based on “attachment theory, behavioral parenting skills, and adult psychopathology” (23). The aim is to improve emotional regulation in caregivers to decrease the risk of child maltreatment (23). Study participants were parents in substance abuse treatment with children aged 2.5 years or younger. The trial-based EE had a time frame of 12 months and analyzed QALY gains in parents, as well as the risk of child abuse through the Brief Child Abuse Potential Inventory (BCAP) (23). Even though quality of life gains in parents are valuable outcomes, they are less relevant regarding child abuse and the wellbeing of the children. Consequently, the analysis of the results focuses on the costs for improvements in BCAP scores. The study resulted in €1,234.8 per BCAP score improvement from the personal social services perspective and €2,037.9 per BCAP score improvement from the societal perspective (23).

Dalziel et al. (25) conducted a trial-based CEA to analyse over six months the *Parents Under Pressure* program in Australia, for methadone-receiving parents of children between 2 and 8 years of age. The authors focus on the Child Abuse Potential Inventory (CAP) to distinguish between abusive and non-abusive parents. The results suggest that the program could be cost-effective in preventing maltreatment with an ICER of €26,527.4 per case of maltreatment avoided (25). Dalziel et al. (25) furthermore report net cost savings ranging from €1.5 million to €6.2 million for 100 families profiting from the *Parents Under Pressure* program.

Both studies seem to be of high quality (>90%) and show results in favor of *Parents Under Pressure* compared to usual care. However, they differ in regard to the age of included participants, the time frame and the reported outcome measure. Barlow et al. (23) did not report individual costs, which affects the transparency of the study. Dalziel et al. (25) had a relatively short follow-up period to assess persisting intervention effects. The conclusions of both studies, however, seem justified considering the given data. *Parents Under Pressure* could be a cost-effective solution to reducing the risk of maltreatment in complex situations involving substance-abusing caregivers.

#### Group family nurse partnership

Barnes et al. (24) provide a trial-based CUA and CEA of the *Group Family Nurse Partnership* program compared to care as usual. As the name indicates, the intervention is conducted by family nurses in a group setting, following young mothers from pregnancy until their children are 1 year old (24). The principles of the intervention are adapted from the *Family Nurse Partnership* intervention. The aim of the program is to provide group sessions to mothers with similar characteristics and generally low educational levels to improve parenting skills and increase infant and maternal health (24). Similarly to the study by Barlow et al. (23), the QALY analysis focused on parents, and consequently the outcome of interest is the risk of child maltreatment. The Adult Adolescent Parenting Inventory (AAPI-2) and the CARE index for maternal sensitivity were used to distinguish between abusive and non-abusive parenting. The intervention showed a low probability for being cost-effective at a cost-effectiveness threshold of €20,000, with an ICER of €130,543 per AAPI-2 score improvement. The study fulfilled all the criteria in assessing the methodological quality, based on the CHEC-extended checklist (100%).

#### Abusive head trauma prevention

Beaulieu et al. (27) study the costs of abusive head trauma (AHT) and provide a CBA of the Period of PURPLE crying program, a prevention program for AHT. The Period of PURPLE crying intervention aims to educate parents about the normality of increased crying of their (healthy) baby in the first few months of their life, termed “period of PURPLE crying” (27, 34). The model-based study is based on the number of AHT cases reported in British Columbia (Canada) between 2002 and 2014 and estimates the lifetime costs of AHT. The cost-benefit estimates are based on a 35% prevention of AHT cases, on the average costs in the study population and the probability of AHT with or without the PURPLE program (27). The 35% are based on a study conducted in British Columbia, where a 35% decrease in AHT hospitalizations was observed after the implementation of the program (34). From a societal perspective, a one Euro investment would result in a savings of €54. From a healthcare perspective, a one Euro investment would result in a savings of €2.9. As the expected costs per child are lower in the intervention group compared to the group not receiving the program, and the effectiveness higher, the intervention is considered dominant.

Another study analyzing a prevention program for AHT has been conducted by Friedman et al. (30) in New Zealand. The intervention consists of the provision of information by maternity nurses on crying in babies and the risks and consequences of shaken baby syndrome, through a leaflet and a video (30). The authors conducted a model-based CUA of a national primary prevention program for AHT compared to no intervention, including lifetime costs. The costs were based on the review of a 5-year cohort and the incidence taken from a 3-year prospective study (30). QALYs were derived from the CHIP study conducted in the Netherlands. For an effectiveness of 5% and a cost of NZ$20 intervention (€12.5) the ICUR would be €4,436.5 cost per QALY saved. The study concludes that a higher effectiveness with reasonable costs would result in the intervention being dominant, saving money per QALY gained (30).

**Figure 2:**
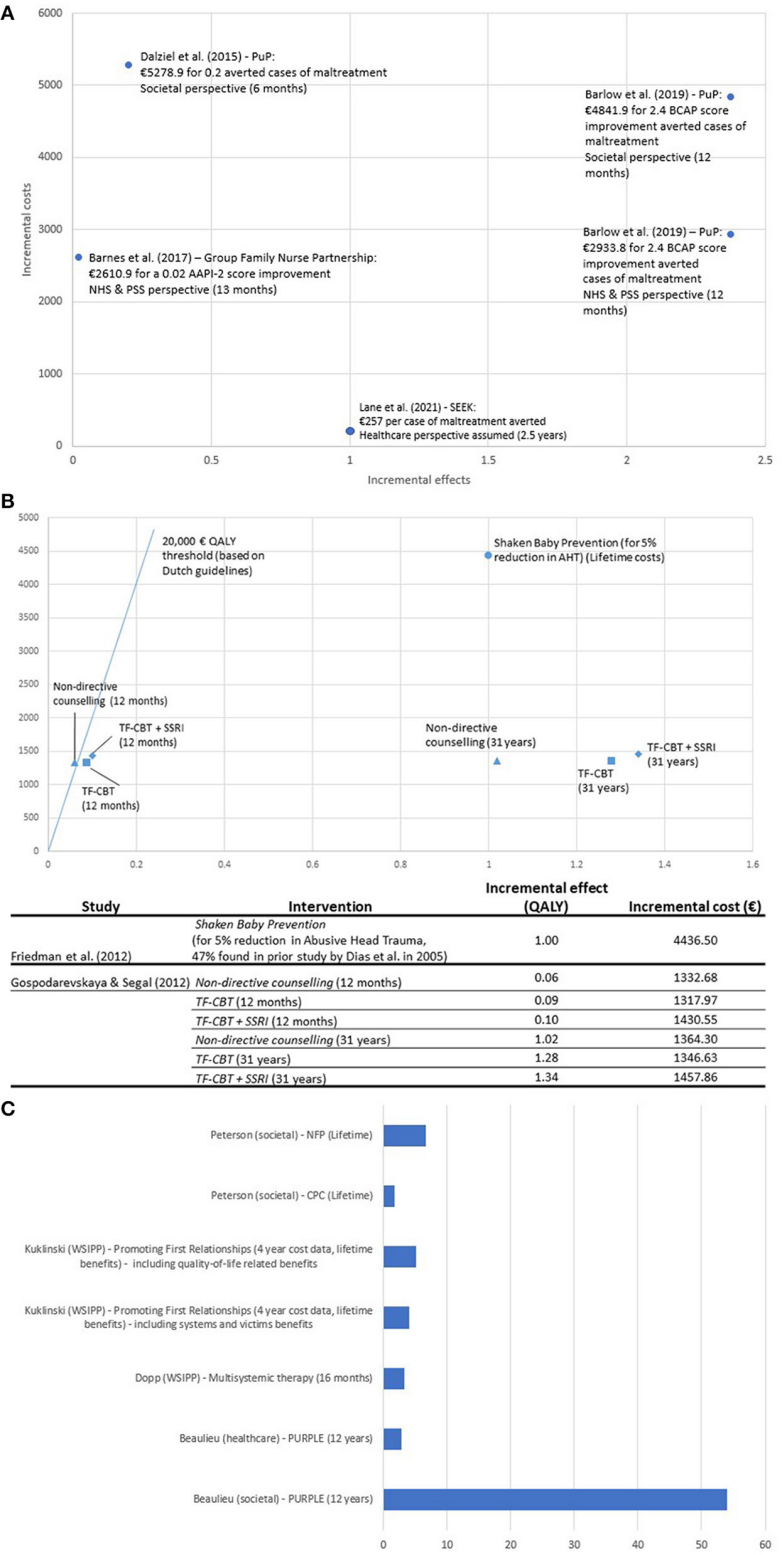
**(A)** Cost-effectiveness plane for studies including a cost-effectiveness analysis (CEA). Block et al. (28) not included in graph due to scale: Block et al. (28): €89,268 for one additional criminal conviction (societal perspective assumed). **(B)** Cost-effectiveness plane for studies including a cost-utility analysis (CUA). **(C)** Results of benefit-cost ratio studies (Cost-savings from a one Euro investment).

The quality of both studies was considered acceptable. Beaulieu et al. (27) did not describe the costs of the intervention in detail. Friedman et al. (30) did not mention the perspective, which had to be assumed. The quality of the study by Friedman et al. (30) was slightly lower as details on the perspective were missing, and not all outcomes were reported. However, based on the results from both studies, preventive interventions for abusive head trauma show a high likelihood of being dominant Consequently, both studies or interventions can be found in the SE quadrant in the cost-effectiveness plane (Figure 2).

#### Promoting first relationships

Kuklinski et al. (32) provide a CBA based on the WSIPP model to determine the cost-effectiveness of *Promoting First Relationships* in households with an open Child Protective Services report of possible abuse or neglect. *Promoting first Relationships* is a home-based intervention for children aged 0 to 5 years old and their caregivers (32). Out-of-home placements were used as a proxy to determine reductions in child abuse and neglect. As child abuse and neglect had to be deducted from out-of-home placements, different values were used for the effect size of monetizable child abuse and neglect benefits (32). Consequently, benefit-cost ratios for different effect sizes of abuse and neglect, as a percentage of the effect size of out-of-home placements, were calculated. For a 20% effect size of CAN compared to out-of-home placements, the authors estimated a benefit-cost ratio (BCR) of €4.13 in scenario 2, including systems and victims benefits, and a BCR of €5.19 in scenario 3, including quality-of-life related benefits. Besides the assumptions on the effect size, which may not be completely accurate, the study showed high quality (97.4%) and the intervention could be cost-effective

#### Safe environment for every kid

Lane et al. (26) provide an analysis of the cost-effectiveness of the *Safe Environment for every Kid* (*SEEK*) in comparison with routine pediatric care over 3 years in children below 6 years old. *SEEK* is embedded in pediatric primary care services and consists of a questionnaire filled out by the parents to assess psychosocial risk factors for child maltreatment. The identified risk factors are then addressed by the primary care provider (26, 35). Based on a trial conducted previously by Dubowitz et al. (36), the cost-effectiveness of *SEEK* was estimated for a population of 29,610 children (26). Even though the quality of the study was acceptable (83.3%), the perspective had to be assumed. Their results include an ICER of €257 per case of maltreatment averted, which is considered cost-effective (26).

#### Child parent centers and nurse-family partnership

Peterson et al. (33) provide estimates of the cost-effectiveness of *Child Parent Centers* (*CPC*) and the *Nurse-Family Partnership* in each US state. To simplify reporting of their results, the estimates of the net present value per avoided case of CAN for the total population were analyzed and adjusted. The *CPC* in preschool resulted in an ICER of only €49,627.1 per averted case of CAN (payer perspective) and savings of €84,211.5 per averted case of CAN (societal perspective). The *CPC* in preschool and school age resulted in an ICER of €45,600.2 per averted case of CAN (payer perspective) and savings of €88,336.6 per averted case of CAN (societal perspective). The *Nurse-Family Partnership* showed savings for both the payer and the societal perspectives, with €24,817.6 and €167,664.3 savings, respectively, per averted case of maltreatment. Furthermore, the BCR for the societal perspective was given, and equals to €1.73 per euro invested for *CPC* and €6.37 for the *Nurse-Family Partnership* (33). Both interventions—*CPC* and *Nurse-Family Partnership*—are considered dominant from a societal perspective as they result in savings per averted case of maltreatment.

### Treatment and diagnosis

#### Multisystemic therapy

Dopp et al. (29) conduct a CBA of *Multisystemic Therapy* for child abuse and neglect. The community-based program is composed of different interventions involving the whole family and their surrounding to identify and address risk factors for maltreatment, treat consequences of maltreatment and prevent further abuse or neglect (29). The intervention was evaluated in families with recently diagnosed physical abuse in children aged 10 to 17. The cost-benefit ratio, based on trial data with a 16-month follow-up, shows that €3.3 could be saved for a 1 Euro investment (cost-effective). The authors reported all relevant aspects required by the CHEC-extended checklist.

#### Trauma-focused cognitive behavioral therapy and non-directive counseling

A model-based CUA was applied over a time frame of 12 months and 31 years, comparing different treatments from an Australian mental health system perspective in children aged 10 years at baseline (31). The CUA includes *Trauma-Focused Cognitive Behavioral Therapy (TF-CBT), TF-CBT in combination with selective serotonin reuptake inhibitors (SSRI)*, and *Non-Directive Supportive Counseling*. *TF-CBT* and *Non-Directive Counseling* are both flexible treatment programs for post-traumatic stress disorder in children consisting of several sessions including the child or the child and caregivers (31). For a 12-month time frame the ICERs for the different interventions were equal to €22,211.3 per QALY gained (*Non-Directive Counseling*), €14,647 per QALY gained (*TF-CBT*) and €14,305.5 per QALY gained *(TF-CBT & SSRI)*. For a 31-year timeframe, they were equal to €1,337.55 per QALY gained (non-directive counseling), €1060.34 per QALY gained *(TF-CBT)* and €1,096.61 per QALY gained *(TF-CBT & SSRI*) (31). *TF-CBT* as well as *TF-CBT in combination with SSRI* were more likely to be cost-effective than *Non-Directive Counseling*.

#### Multiple interviews

Block et al. (28) conducted a model-based CEA of *Multiple Interviews* in the diagnosis of possible sexual abuse in children compared to the usual interviewing procedure. The outcome of interest was the number of additional convictions based on a 6.1% increase in the likelihood of criminal convictions (28). The ICER given in the study is equal to €89,268.6 per additional criminal conviction, which the authors consider to be acceptable regarding the high costs of CAN and the number of cases that may be prevented through one additional conviction (28). Based on the comparatively low study quality (58.3%) and the methodological difficulties of measuring additional criminal convictions, no conclusion can be drawn whether *Multiple Interviews* are cost-effective in comparison with single interviews.

### Cost-effectiveness plane

In Figures 2A–C, the results are summarized in cost-effectiveness planes (Figures 2A, B) and one additional graph for the studies including a BCR (Figure 2C). Figure 2A focuses on studies including a CEA or clinical outcomes, while Figure 2B focuses on CUA studies using QALY as outcomes. The cost-effectiveness planes visually presents the results from different EEs, based on their incremental effects and incremental costs. Due to the heterogeneity of individual studies, several graphs were created. As the outcomes are varying, the position of different studies or interventions may be to some extent arbitrary, but the figures provides first insight into the cost-effectiveness of different interventions. As can be seen, most included studies are situated in the north-eastern (NE) quadrant. This means that they show beneficial effects for additional costs, which requires a decision to be taken based on a willingness-to-pay threshold for a certain outcome. Four interventions seem particularly likely to be cost-effective, or dominant, as they have high incremental effects for lower costs (27, 30, 33). Dominant interventions can be found in the south-eastern (SE) quadrant. On the other hand, two interventions seem unlikely to be cost-effective compared to other interventions, as they show low effects for high incremental costs (24, 28).

## Discussion

The aim of this review was to assess the current evidence on economic evaluations of interventions aimed at the prevention or treatment of child abuse and neglect in high-income countries.

Only a small number of economic evaluations focusing on child abuse and neglect have been retrieved and fulfilled the inclusion criteria. This highlights the need for further studies analyzing the cost-effectiveness of interventions relating to child abuse and neglect. Even though a sensitive search strategy was applied, only 11 economic evaluations were eligible. Most studies are considered to be cost-effective, which may be partly due to publication bias. Due to the heterogeneity of the studies, the results were not pooled as they are incomparable to a large extent.

The quality of the economic evaluations was high, with an average of 89.21%. Only one study scored below 80% on the CHEC-extended checklist. The applied methodology varied considerably between studies in regard to the type of economic evaluations performed and the type of outcomes measured. Most quality concerns were related to the description of the chosen perspective, the included costs, and discounting procedures.

The results have shown that most of the included studies were cost-effective in tackling child abuse and neglect. The *Parents Under Pressure* program has shown evidence of improving outcomes at lower costs than the comparators in caregivers of children aged 2.5 years or younger in the UK and in children aged 2 to 8 years in Australia (23, 25). Two studies have shown that interventions focusing on the prevention of abusive head trauma or shaken baby syndrome show a high likelihood of leading to beneficial outcomes at lower costs than the comparators. In other words, they show a high likelihood of being dominant. *The Period of PURPLE crying* implemented in Canada was compared to no program and showed an acceptable return on investment and cost-savings (27). Another basic shaken baby prevention program in New Zealand has shown low costs or even cost savings per QALY gain (30). *Child Parent Centers* and *Family-Nurse Partnership* also showed a high likelihood of being dominant, i.e., resulting in cost savings per case of maltreatment averted from a societal perspective (33).

Only one included study focused on an intervention implemented in primary care services. The implementation of *Safe Environment for Every Kid* in a population of around 30,000 children showed low costs per case of maltreatment averted (26). The *Group Family Nurse Partnership* did not show evidence of being cost-effective (24). The interventions focusing on the treatment of children who have experienced child abuse and neglect were also found to be cost-effective (29, 31). *Cognitive Behavioral Therapy* focused on trauma seems to be more cost-effective than non-directive counseling. The addition of selective serotonin reuptake inhibitors to *Trauma-Focused Cognitive Behavioral Therapy* may provide even lower costs per QALY gains (31). However, the use of selective serotonin reuptake inhibitors in children may also include other risks, not included in the economic evaluation. *Multisystemic Therapy* has shown an acceptable benefit-cost ratio (29). Based on the comparatively low study quality and the methodological difficulties of measuring additional criminal convictions, no definite conclusion can be drawn about whether multiple interviews are cost-effective in comparison with single interviews (28).

This review has several strengths. Following the PRISMA framework and guidelines on conducting a systematic review of economic evaluations is expected to ensure the methodological quality of this review. Furthermore, a PROSPERO protocol was developed before conducting the review. In addition, the data extraction and quality assessment was checked by two researchers independently.

The review is, however, subject to several limitations. Publication bias has not been estimated. No gray literature was included, and studies published in languages other than German, French or English were excluded. Due to the heterogeneity of retrieved studies and varying terminologies and methodologies, the selection of articles may have been arbitrary. While the selection of studies has been done by two researchers independently, no intercoder agreement score was determined. In addition, transferability was not assessed, as the review does not focus on the implementation of interventions in one particular country or setting.

Several shortcomings of individual economic evaluations in the field of child abuse and neglect were identified. There appears to be a need for more standardized reporting methods, as the results of the review depend strongly on methodological choices and the reporting quality of included studies. Dalziel et al. (25) and Barlow et al. (23), for example, include different methods for discriminating between abusive and non-abusive parents. Barlow et al. (23) report the cost per Brief Child Abuse Potential Inventory (BCAP) score improvement, while Dalziel et al. (25) report the cost per prevented case of maltreatment based on Child Abuse Potential Inventory (CAP) cut-off values and the respective risks of maltreatment. Preferably, studies applying either the CAP or the BCAP, which correlate strongly, should report the same outcome. The benefits and disadvantages of using BCAP or CAP scores, or cut-off points to estimate the number of prevented cases of maltreatment should be further analyzed. Standardized reporting of the outcome would allow further comparisons and pooling of results. Other methods used to assess the number of maltreatment cases or prevented maltreatment cases include the Adult Adolescent Parenting Inventory or the Conflict Tactics Scales: Parent-Child version (24, 26). These methods may yield different results and reflect prevented child abuse and neglect cases more or less accurately. The assessment of QALY in children is also subject to several limitations (37).

Possible influences could be the sample sizes, the handling of missing data or the models used, which may also have a large influence on the effectiveness and cost-effectiveness results. In addition, the included costs and time frames vary, highlighting the lack of a common methodology for analyzing cost-effectiveness of interventions for abuse and neglect. Therefore, the results of studies have not been pooled and comparisons between studies should be made with caution.

Furthermore, there is no common terminology applied in research on child abuse and neglect. A highly sensitive search strategy was applied to ensure the inclusion of relevant studies. However, as there are no strictly defined boundaries on what should be considered abuse and neglect, it is difficult to determine which studies to include. Furthermore, even if boundaries are well-defined, it is difficult to accurately measure the prevalence or number of maltreatment cases in a certain population.

The most recent identified reviews including economic evaluations of child abuse and neglect interventions reported similar limitations. Peterson and Kearns (16) mention the need for better reporting standards to increase comparability between economic evaluations of violence prevention interventions. El-Banna et al. (15) furthermore highlight the lack of standardized outcome measures and cost-effectiveness threshold in children's social care interventions. In addition, the time frame of the economic evaluations is often too short to include long-term costs and effects of the interventions (15). Based on the identified limitations, El-Banna et al. (15) developed ten recommendations for future systematic reviews of economic evaluations children's social care interventions.

Due to the mentioned limitations of the field of study and of the studies included, some arbitrary decisions had to be taken while developing and applying the inclusion and exclusion criteria. This study included articles focusing on the four main types of child abuse and neglect, including abusive head trauma. Intimate partner violence was not included as it does not necessarily lead to maltreatment. Furthermore, studies had to focus specifically on children at risk of abuse or neglect or children who experienced abuse or neglect. Economic evaluations of studies focusing on broader outcomes with possible effects on abuse and neglect did not meet the inclusion criteria. Children or caregivers with mental health disorders were not included. Other studies that did not meet the inclusion criteria but may provide additional information include, among others: Aas et al. (38), Dijkstra et al. (39), Johnson-Motoyama et al. (40), Lynch et al. (41) and Reynolds et al. (42). These studies were excluded for different reasons. Aas et al. (38) focused on children who experienced a trauma which does not exclusively focus on traumas related to abuse and neglect. Johnson-Motoyama et al. (40) focused on out-of-home placements in substance-affected families, which is not necessarily linked on abuse and neglect. Reynolds et al. (42) also did not focus specifically on abuse and neglect related outcomes. Lynch et al. (41) included children in foster care and permanent placements as main outcome, which does not reflect abuse and neglect. The outcome measure in Dijkstra et al. (39) was considered insufficient to measure prevented cases of maltreatment.

Regarding the generalizability of findings, it should be kept in mind that a study that has been found to be cost-effective in a specific setting and population is not necessarily cost-effective in another setting and population. The generalizability of the results presented in this study are limited to high-income countries. Primary care and childcare services vary across countries. Transferability analysis should be performed to ensure that an intervention will remain cost-effective in a different setting. Therefore, one should have a clear idea about the structure of childcare services in the country of interest and the basic level of care. For example, abusive head trauma prevention interventions which have been found to be cost-effective may already be part of the basic care provided to parents in other countries. Otherwise, it might be a cost-effective prevention measure to reduce abusive head traumas in maternal care or primary, pediatric care. Treatment interventions might be integrated into existing childcare services. To determine the transferability of the economic evaluation, the Welte checklist may be used, including general checkout criteria, methodological characteristics, healthcare system characteristics and population characteristics (20). To further investigate the transferability, the PIET-t model in “Models of Child Health Appraised” may serve as a helpful tool for assessing similarities between childcare systems and identifying possible barriers to implementation (43).

Based on the results of the review and the identified limitations, several recommendations for policy and future research will be made. The presented results are expected to provide insight to policymakers in high-income countries on financially sustainable possibilities to tackle child abuse and neglect. Interventions to prevent abusive head trauma through simple educational means (e.g., fact sheets) have shown high cost-effectiveness. As they show considerable effects for low costs and efforts, they are expected to be cost-effective in varying settings. Furthermore, home-based, individualized interventions to prevent maltreatment may be of interest for policymakers. While the priority should be on preventing abuse and neglect, the treatment options have shown promising results for being cost-effective.

To overcome current shortcomings, expertise from different fields is required when conducting economic evaluations in the field of child abuse and neglect and should be integrated into the development and evaluation of interventions. A common methodology would strongly benefit future reviews and economic evaluations of relevant interventions. More research is needed to determine the most accurate and useful outcome measure in economic evaluations on child abuse and neglect. Developing a common methodology and outcome measure will allow further comparisons or pooling of data in a meta-analysis. Additionally, a common methodology and outcome measure will facilitate the development and application of strict inclusion and exclusion criteria for future systematic reviews. Spillover effects should be estimated in future research as child abuse and neglect interventions impact various dimensions of a caregiver's and/or children's life as well as the people around them. Neglecting spillover effects such as costs of informal care and benefits to family members, may result in an underestimation of the benefits of child abuse and neglect interventions (44).

Further research is required to determine which intervention shows the most favorable cost-effectiveness in different settings. Future researchers should adhere to the guidelines established by the Professional Society for Health Economics and Outcomes Research (ISPOR). Furthermore, there are several reporting guidelines for economic evaluations, such as the recently updated Consolidated Health Economic Evaluation Reporting Standards (CHEERS), to ensure all relevant study aspects are reported (45). Using recognized guidelines ensures that all relevant study aspects will be reported, facilitating future reviews and the development of replicable methodologies.

## Conclusion

This study provides an overview of economic evaluations of preventive, diagnostic and treatment interventions related to child abuse and neglect in high-income countries. The results show that little research has been done in this field, but the evaluated interventions have a high potential for cost-effectiveness, especially individualized home- or community-based interventions and educational interventions. The transferability should, however, be assessed before implementing the interventions in a new setting. Future research could benefit from a more strictly defined terminology for child abuse and neglect, and from clear boundaries on which caregiver practices are considered to be abuse or neglect. Furthermore, a common methodology could increase the comparability of interventions focusing on child abuse and neglect.

## Data availability statement

The original contributions presented in the study are included in the article/Supplementary material, further inquiries can be directed to the corresponding author.

## Author contributions

TK, IW, and SE were involved throughout the conception and the development of the design of the study. BB provided expert opinion on child abuse and neglect, methodological guidance (e.g., search strategy, definitions, inclusion/exclusion criteria), as well as important aspects to consider in the discussion and conclusion. GM and SE provided guidance on the design of systematic reviews of economic evaluations and the reporting and interpretation of results. TK conducted the analysis and wrote the first draft of the article and integrated the feedback. IW independently conducted certain steps of the analysis. All authors approved the final article for publication, critically reviewed the manuscript, and provided feedback.

## Supplementary material

Supplementary Appendix A Prospero Protocol.

Supplementary Appendix B Search strategy.

Supplementary Appendix C CHEC-extended checklist.
