# Supplementary material for: Economic evaluations of interventions focusing on child abuse and neglect in high-income countries: a systematic review
Source: Front Psychiatry. 2023 Jun 21;14:1031037. doi: 10.3389/fpsyt.2023.1031037 (PMC10320140; doi:10.3389/fpsyt.2023.1031037)
Supplement: Supplementary Appendix A — Prospero Protocol. [file Data_Sheet_1.docx]

**Appendix A: PROSPERO Protocol**

The protocol has been updated in July 2021 and is still being assessed. The original protocol can be found under the following link: <https://www.crd.york.ac.uk/prospero/display_record.php?ID=CRD42021248485>


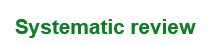


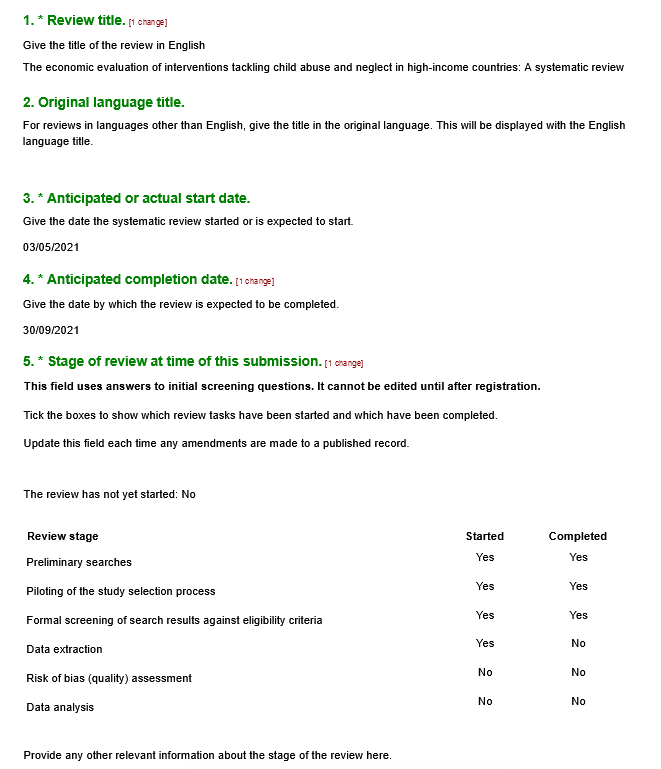


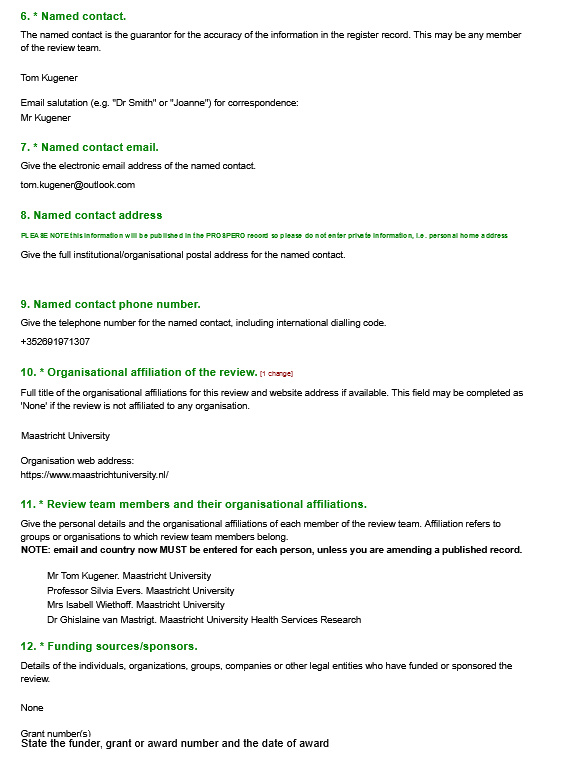


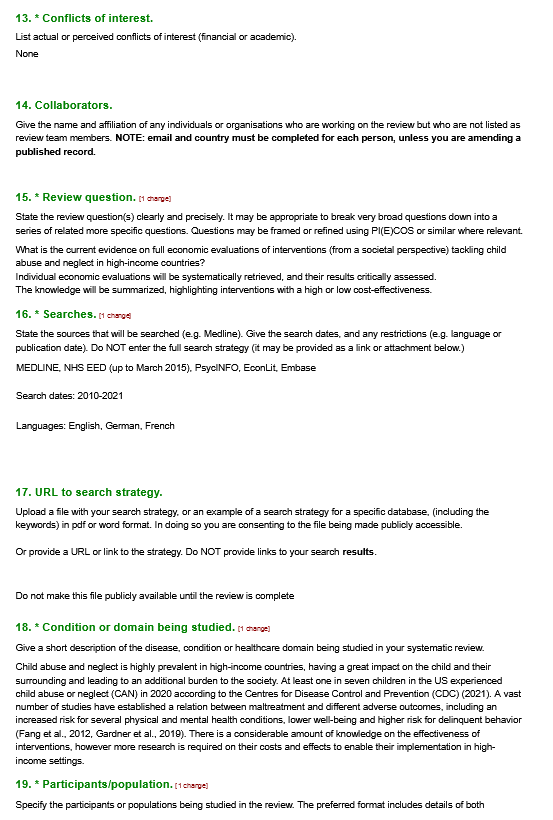


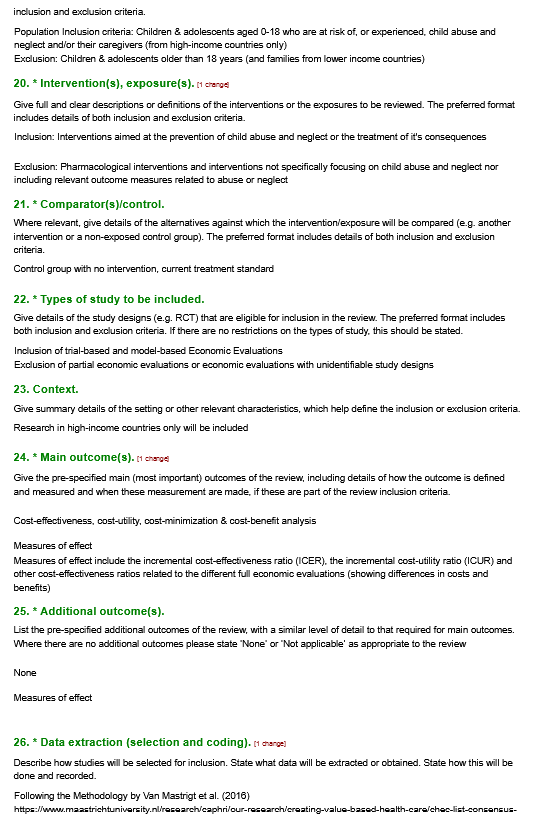


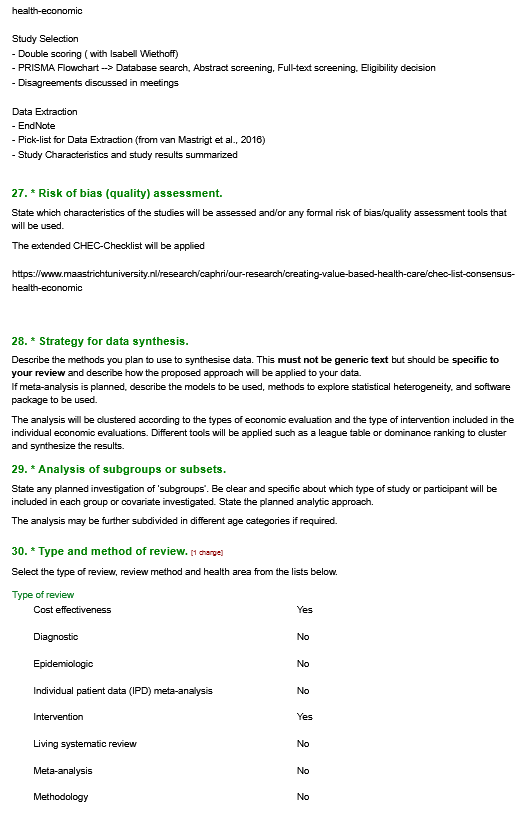


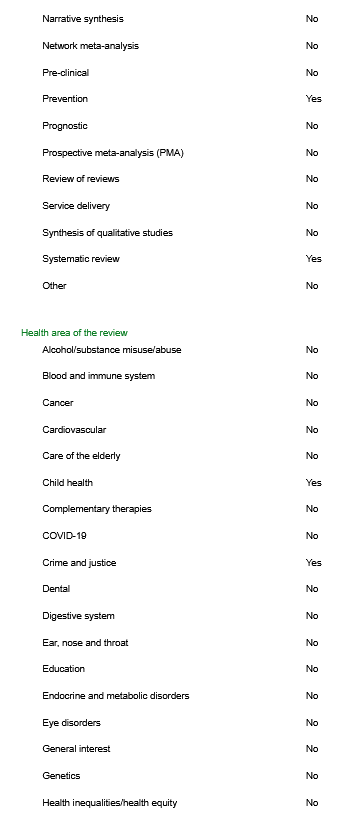


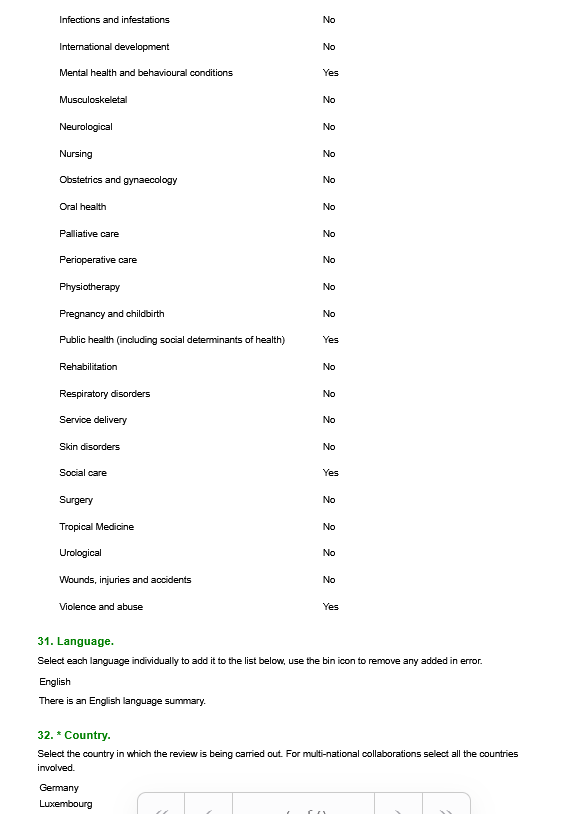


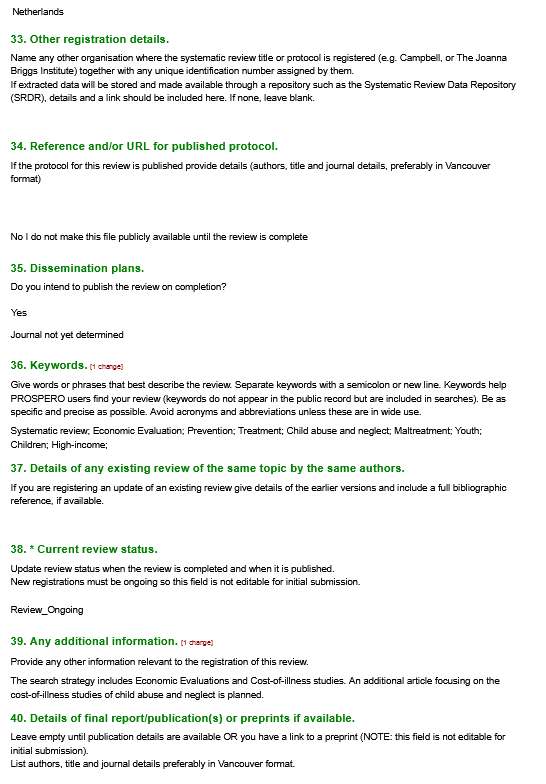


# **Appendix B: Search Strategy**

**Table 3.**
Number of Search Results

| Database | Number of Results | Date of Search | Without duplicates |
| --- | --- | --- | --- |
| MEDLINE (OVID) | 1273 | 04.05.2021 | 1261 |
| EMBASE (OVID) | 2584 | 04.05.2021 | 1448 |
| PSYCINFO (EBSCOHOST) | 1679 | 04.05.2021 | 1298 |
| ECONLIT (EBSCOHOST) | 247 | 04.05.2021 | 210 |
| NHS EED | 82 | 04.05.2021 | 69 |
|  |  |  |  |
| TOTAL | 5865 |  | 4286 |
| AFTER DEDUPLICATION | 5865 - 1579 = 4286 |  |  |

**Table 4.**
MEDLINE Search Strategy

| **#** | **Search Filters** | **# of Results** |
| --- | --- | --- |
| **Youth** | | |
| 1* | (teen* OR youth* OR adolescen* OR juvenile* OR (young ADJ2 (adult* OR person* OR individual* OR people* OR population* OR man OR men OR wom#n)) OR youngster* OR first-grader* OR second-grader* OR third-grader* OR fourth-grader* OR fifth-grader* OR sixth-grader* OR seventh-grader* OR highschool* OR college* OR ((secondary OR high*) ADJ2 (school* OR education))).ti,ab. OR adolescent/ OR young adult/ | 2,766,144 |
| 2* | (child* OR stepchild* OR step-child* OR kid OR kids OR girl OR girls OR boy OR boys OR teen* OR youth* OR youngster* OR adolescent* OR adolescence OR preschool* OR pre-school* OR kindergarten* OR school* OR juvenile* OR minors OR p?ediatric* OR PICU).ti,ab. OR exp child/ | 2,682,453 |
| **Economic Evaluation** | | |
| 3** | Economics/ or exp "Costs and Cost Analysis"/ or Economics, Nursing/ or Economics, Medical/ or Economics, Pharmaceutical/ or exp Economics, Hospital/ or Economics, Dental/ or exp "Fees and Charges"/ or exp Budgets/ or budget*.ti,ab,kf. or (economic* or cost or costs or costly or costing or price or prices or pricing or pharmacoeconomic* or pharmaco-economic* or expenditure or expenditures or expense or expenses or financial or finance or finances or financed).ti,kf. or (economic* or cost or costs or costly or costing or price or prices or pricing or pharmacoeconomic* or pharmaco-economic* or expenditure or expenditures or expense or expenses or financial or finance or finances or financed).ab. /freq=2 or (cost* adj2 (effective* or utilit* or benefit* or minimi* or analy* or outcome or outcomes)).ab,kf. or (value adj2 (money or monetary)).ti,ab,kf. or exp models, economic/ or economic model*.ab,kf. or markov chains/ or markov.ti,ab,kf. or monte carlo method/ or monte carlo.ti,ab,kf. or exp Decision Theory/ or (decision* adj2 (tree* or analy* or model*)).ti,ab,kf. | 651,089 |
| 4*** | ((cost adj2 illness) or (cost* adj2 disease) or ("spending*" or "healthcare use" or "healthcare usage" or "health care use" or "health care usage" or "healthcare resource utili?ation" or "health care resource utili?ation" or "health care resource use" or "healthcare resource use" or "resource utili?ation" or "productivity loss*" or "disease burden" or "economic burden" or "societal burden" or "socio-economic burden")).ti,ab,kf. | 53,655 |
| **Child abuse and neglect** | | |
| 5**** | (child protection or social service or social care or childrens services or child protection services or family support services).ti,ab. Or ((child or children) adj2 (protection or welfare or service* or advocacy)).ti,ab. Or ((family or youth or welfare) adj1 (service* or support)).ti,ab. Or ((neglect or neglected or abuse or abused or violence or exploit* or maltreatment) adj3 (child or children or adolescen* or teen* or youth)).ti,ab. Or (parent* adj2 (capacity or problem* or disability* or disable* or illness or absent* or absence or program*)).ti,ab. Or (family adj2 (dysfunction or stress or preservation)).ti,ab.  (<https://www.sciencedirect.com/science/article/pii/S0190740920322866> ) | 54,674 |
| 6 | 1 OR 2 | 4,136,383 |
| 7 | 3 OR 4 | 678,532 |
| 8 | 5 AND 6 AND 7 | 2,895 |
| 9 | limit 8 to (humans and yr="2010 - 2021") | 1,315 |
| 10 | Limit 9 to (editorial or guideline or letter) | 15 |
| 11 | 9 NOT 10 | 1,300 |
| 12 | limit 11 to (english or french or german) | **1,273** |

Note. Different search filters for the same concept combined by Boolean operator “OR” and different concepts combined by Boolean operator “AND”.
* Adapted from the Candadian Health Libraries Association (43)
** Adapted from CADTH database search filters [Internet]. Ottawa: CADTH; 2021. <https://www.cadth.ca/strings-attached-cadths-database-search-filters>
***Developed in collaboration with Wiethoff (2021)
**** Search filter adapted from El-Banna, Petrou (15)

**Table 5.**EMBASE Search Strategy

| **#** | **Search Filters** | **# of results** |
| --- | --- | --- |
| **Youth** | | |
| 1* | (teen* OR youth* OR adolescen* OR juvenile* OR (young ADJ2 (adult* OR person* OR individual* OR people* OR population* OR man OR men OR wom#n)) OR youngster* OR first-grader* OR second-grader* OR third-grader* OR fourth-grader* OR fifth-grader* OR sixth-grader* OR seventh-grader* OR highschool* OR college* OR ((secondary OR high*) ADJ2 (school* OR education))).ti,ab. OR exp adolescent/ OR exp adolescence/ OR young adult/ | 2,484,741 |
| 2* | (child* OR stepchild* OR step-child* OR kid OR kids OR girl OR girls OR boy OR boys OR teen* OR youth* OR youngster* OR adolescent* OR adolescence OR preschool* OR pre-school* OR kindergarten* OR school* OR juvenile* OR minors OR p?ediatric* OR PICU).ti,ab. OR exp child/ | 3,829,922 |
| **Economic Evaluation** | | |
| 3** | Economics/ or Cost/ or exp Health Economics/ or Budget/ or budget*.ti,ab,kw. or (economic* or cost or costs or costly or costing or price or prices or pricing or pharmacoeconomic* or pharmaco-economic* or expenditure or expenditures or expense or expenses or financial or finance or finances or financed).ti,kw. or (economic* or cost or costs or costly or costing or price or prices or pricing or pharmacoeconomic* or pharmaco-economic* or expenditure or expenditures or expense or expenses or financial or finance or finances or financed).ab. /freq=2 or (cost* adj2 (effective* or utilit* or benefit* or minimi* or analy* or outcome or outcomes)).ab,kw. or (value adj2 (money or monetary)).ti,ab,kw. or Statistical Model/ or economic model*.ab,kw. Or Probability/ or markov.ti,ab,kw. or monte carlo method/ or monte carlo.ti,ab,kw. or Decision Theory/ or Decision Tree/ or (decision* adj2 (tree* or analy* or model*)).ti,ab,kw. | 1,736,116 |
| 4*** | ((cost adj2 illness) or (cost* adj2 disease) or ("spending*" or "healthcare use" or "healthcare usage" or "health care use" or "health care usage" or "healthcare resource utili?ation" or "health care resource utili?ation" or "health care resource use" or "healthcare resource use" or "resource utili?ation" or "productivity loss*" or "disease burden" or "economic burden" or "societal burden" or "socio-economic burden")).ti,ab,kw. | 103,979 |
| **Child abuse and neglect** | | |
| 5**** | (child protection or social service or social care or childrens services or child protection services or family support services).ti,ab. Or ((child or children) adj2 (protection or welfare or service* or advocacy)).ti,ab. Or ((family or youth or welfare) adj1 (service* or support)).ti,ab. Or ((neglect or neglected or abuse or abused or violence or exploit* or maltreatment) adj3 (child or children or adolescen* or teen* or youth)).ti,ab. Or (parent* adj2 (capacity or problem* or disability* or disable* or illness or absent* or absence or program*)).ti,ab. Or (family adj2 (dysfunction or stress or preservation)).ti,ab.  (<https://www.sciencedirect.com/science/article/pii/S0190740920322866> ) | 78,188 |
| 6. | 1 OR 2 | 4,927,865 |
| 7. | 3 OR 4 | 1,785,915 |
| 8. | 5 AND 6 AND 7 | 5,763 |
| 9. | limit 8 to (human and yr="2010 - 2021") | 3,344 |
| 10. | limit 9 to (english or french or german) | 3,295 |
| 11. | Limit 8 to (conference abstract or editorial or letter) | 711 |
| 12. | 10 NOT 11 | **2,584** |

Note. Different search filters for the same concept combined by Boolean operator “OR” and different concepts combined by Boolean operator “AND”.
* Adapted from the Candadian Health Libraries Association (43)
** Adapted from CADTH database search filters [Internet]. Ottawa: CADTH; 2021. <https://www.cadth.ca/strings-attached-cadths-database-search-filters>
***Developed in collaboration with Wiethoff (2021)
**** Search filter adapted from El-Banna, Petrou (15)

**Table 6.**
PsycInfo Search Strategy

| **#** | **Search Filters** | **# of results** |
| --- | --- | --- |
| **Youth** | | |
| 1* | AB (teen OR youth OR adolescen* OR young adult* OR young person* OR young individual* OR young people* OR young population* OR young man OR young men OR young wom#n OR youngster* OR first-grader* OR second-grader* OR third-grader* OR fourth-grader* OR fifth-grader* OR sixth-grader* OR seventh-grader* OR highschool* OR college* OR secondary school OR high school*) | 543,074 |
| 2* | AB (child* OR stepchild* OR step-child* OR kid OR kids OR girl OR girls OR boy OR boys OR teen* OR youth* OR youngster* OR adolescent* OR adolescence OR preschool* OR pre-school* OR kindergarten* OR school* OR juvenile* OR minors OR p?ediatric* OR PICU) | 1,093,240 |
| **Economic Evaluation** | | |
| 3** | MA economics OR MA cost OR MA health economics OR MA budget (17,767)  Or  TI (economic* or cost or costs or costly or costing or price or prices or pricing or pharmacoeconomic* or pharmaco-economic* or expenditure or expenditures or expense or expenses or financial or finance or finances or financed) (42,329)  Or  AB (cost* N2 (effective* or utilit* or benefit* or minimi* or analy* or outcome or outcomes)) (27,747)  Or AB (value N2 (money or monetary) or Statistical Model or economic model* Or Probability or markov or monte carlo method or monte carlo or Decision Theory or Decision Tree or decision* N2 (tree* or analy* or model*)) (84,853) | 354,235 |
| 4 *** | AB (cost N2 (illness or disease) or "healthcare utili*" or burden N2 (illness or economic or disease)) | 8,759 |
| **Child abuse and neglect** | | |
| 5**** | AB (child protection or social service or social care or childrens services or child protection services or family support services) (54,307)  Or  AB ((child or children) N2 (protection or welfare or service* or advocacy)) (22,469)  Or  AB ((family or youth or welfare) N1 (service* or support) Or (neglect or neglected or abuse or abused or violence or exploit* or maltreatment) N3 (child or children or adolescen* or teen* or youth)) (56,343)  Or  AB (parent* N2 (capacity or problem* or disability* or disable* or illness or absent* or absence or program*)) (16,494)  Or  AB (family N2 (dysfunction or stress or preservation)) (6186) | 124,877 |
| 6 | S1 or S2 | 1,239,616 |
| 7 | S3 or S4 | 159,990 |
| 8 | S5 | 124,925 |
| 9 | 6 AND 7 AND 8 | 3,133 |
| 10 | **Limiters** - Publication Year: 2010-2021; Language: English, French, German; Population Group: Human | **1,679** |
| 11 | Limiters - Document Type: Abstract Collection, Column/Opinion, Editorial, Letter | **14** |
| 10 NOT 11 |  | **1,665** |

Note. Different search filters for the same concept combined by Boolean operator “OR” and different concepts combined by Boolean operator “AND”.
* Adapted from the Candadian Health Libraries Association (43)
** Adapted from CADTH database search filters [Internet]. Ottawa: CADTH; 2021. <https://www.cadth.ca/strings-attached-cadths-database-search-filters>
***Developed in collaboration with Wiethoff (2021)
**** Search filter adapted from El-Banna, Petrou (15)

**Table 7.**
EconLit Search Strategy

| **#** | **Search Filters** | **# of results** |
| --- | --- | --- |
| **Youth** | | |
| 1* | AB (teen OR youth OR adolescen* OR young adult* OR young person* OR young individual* OR young people* OR young population* OR young man OR young men OR young wom#n OR youngster* OR first-grader* OR second-grader* OR third-grader* OR fourth-grader* OR fifth-grader* OR sixth-grader* OR seventh-grader* OR highschool* OR college* OR secondary school OR high school*) | 24,858 |
| 2* | AB (child* OR stepchild* OR step-child* OR kid OR kids OR girl OR girls OR boy OR boys OR teen* OR youth* OR youngster* OR adolescent* OR adolescence OR preschool* OR pre-school* OR kindergarten* OR school* OR juvenile* OR minors OR p?ediatric* OR PICU) | 61,471 |
| **Economic Evaluation** | | |
| 3** | MA economics OR MA cost OR MA health economics OR MA budget (459)  OR  AB (economic* or cost or costs or costly or costing or price or prices or pricing or pharmacoeconomic* or pharmaco-economic* or expenditure or expenditures or expense or expenses or financial or finance or finances or financed) (306,181)  OR  AB (cost* N2 (effective* or utilit* or benefit* or minimi* or analy* or outcome or outcomes)) (19,319)  OR AB (value N2 (money or monetary)) or Statistical Model or economic model* Or Probability or markov or monte carlo method or monte carlo or Decision Theory or Decision Tree or (decision* N2 (tree* or analy* or model*)) (77,259) | 380,702 |
| 4 *** | AB ((cost N2 (illness or disease) or "healthcare utili*" or (burden N2 (illness or economic or disease)) | 1,115 |
| **Child abuse and neglect** | | |
| 5**** | AB (child protection or social service or social care or childrens services or child protection services or family support services) (3,570)  OR  AB ((child or children) N2 (protection or welfare or service* or advocacy)) (807)  OR  AB ((family or youth or welfare) N1 (service* or support)) Or ((neglect or neglected or abuse or abused or violence or exploit* or maltreatment) N3 (child or children or adolescen* or teen* or youth)) (1,424)  OR  AB (parent* N2 (capacity or problem* or disability* or disable* or illness or absent* or absence or program*)) (310)  OR  AB (family N2 (dysfunction or stress or preservation)) (68) | 5,486 |
| 6 | S1 or S2 | 70,825 |
| 7 | S3 or S4 | 381,208 |
| 8 | S5 | 5,486 |
| 9 | 6 AND 7 AND 8 | 442 |
| 10 | **Limiters** - Published Date: 20100101-20211231 | **247** |

Note. Different search filters for the same concept combined by Boolean operator “OR” and different concepts combined by Boolean operator “AND”.
* Adapted from the Candadian Health Libraries Association (43)
** Adapted from CADTH database search filters [Internet]. Ottawa: CADTH; 2021. <https://www.cadth.ca/strings-attached-cadths-database-search-filters>
***Developed in collaboration with Wiethoff (2021)
**** Search filter adapted from El-Banna, Petrou (15)

**Table 8.**
NHS EED Search Strategy

| **#** | **Search Filters** | **# of results** |
| --- | --- | --- |
| **Youth** | | |
| 1 | (MeSH DESCRIPTOR Adolescent EXPLODE ALL TREES) IN NHSEED | 2,214 |
| 2 | (MeSH DESCRIPTOR Young Adult EXPLODE ALL TREES) IN NHSEED | 1,046 |
| 3 | (MeSH DESCRIPTOR Child EXPLODE ALL TREES) IN NHSEED | 1,680 |
| **Child abuse and neglect** | | |
| 4* | (child protection or social service or social care or childrens services or child protection services or family support services) Or ((child or children) adj2 (protection or welfare or service* or advocacy)) Or ((family or youth or welfare) adj1 (service* or support)) Or ((neglect or neglected or abuse or abused or violence or exploit* or maltreatment) adj3 (child or children or adolescen* or teen* or youth)) Or (parent* adj2 (capacity or problem* or disability* or disable* or illness or absent* or absence or program*)) Or (family adj2 (dysfunction or stress or preservation)) IN NHS EED | 669 |
| 5 | 1 or 2 or 3 | 3,297 |
| 6 | 4 AND 5 | 173 |
| 7 | * IN NHSEED FROM 2010 TO 2021 |  |
| 8 | 6 AND 7 | **82** |

Note. Different search filters for the same concept combined by Boolean operator “OR” and different concepts combined by Boolean operator “AND”.
* Search filter adapted from El-Banna et al. (2021)

# **Appendix C**

**Table 9.**CHEC-extended Checklist

| **#** | **Question** | **YES** | **SO*** | **NO** |
| --- | --- | --- | --- | --- |
| Q1 | Is the study population clearly described? |  |  |  |
| Q2 | Are competing alternatives clearly described? |  |  |  |
| Q3 | Is a well-defined research question posed in answerable form? |  |  |  |
| Q4 | Is the economic study design appropriate to the stated objective? (Always yes otherwise exclude) |  |  |  |
| Q5 | Are the structural assumptions and the validation methods of the model properly reported? |  |  |  |
| Q6 | Is the chosen time horizon appropriate in order to include relevant costs and consequences? |  |  |  |
| Q7 | Is the actual perspective chosen appropriate? |  |  |  |
| Q8 | Are all important and relevant costs for each alternative identified? |  |  |  |
| Q9 | Are all costs measured appropriately in physical units? |  |  |  |
| Q10 | Are costs valued appropriately? |  |  |  |
| Q11 | Are all important and relevant outcomes for each alternative identified? (Type of outcomes e.g., QALYs would be optimal) |  |  |  |
| Q12 | Are all outcomes measured appropriately? |  |  |  |
| Q13 | Are outcomes valued appropriately? (Only applicable for CBA and CUA) |  |  |  |
| Q14 | Is an appropriate incremental analysis of costs and outcomes of alternatives performed? |  |  |  |
| Q15 | Are all future costs and outcomes discounted appropriately? |  |  |  |
| Q16 | Are all important variables, whose values are uncertain, appropriately subjected to sensitivity analysis? |  |  |  |
| Q17 | Do the conclusions follow from the data reported? |  |  |  |
| Q18 | Does the study discuss the generalizability of the results to other settings and patient/client groups? |  |  |  |
| Q19 | Does the article/report indicate that there is no potential conflict of interest of study researcher(s) and funder(s)? (transparency) |  |  |  |
| Q20 | Are ethical and distributional issues discussed appropriately? |  |  |  |

* Suboptimal
